# Using Phantomless QCT for evaluating BMD evolution in maintenance hemodialysis patients

**DOI:** 10.1038/s41598-025-07025-2

**Published:** 2025-07-01

**Authors:** Yuwen Shen, Qing Hua, Xinyu Pan, Ping Xie, Lianwei Zhang, Linhe Wu, Sitong Yang, Wen Ren, Kefu Liu

**Affiliations:** 1 Department of Medical Imaging, The Affiliated Suzhou Hospital of Nanjing Medical University, Gusu School of Nanjing Medical University, No.242, GuangJi Road, Suzhou, 215008 Jiangsu China; 2 Department of Nephrology, The Affiliated Suzhou Hospital of Nanjing Medical University, Gusu School of Nanjing Medical University, Suzhou, 215008 Jiangsu China

**Keywords:** Phantom-less quantitative computed tomography, Maintenance hemodialysis, Bone mineral density, Hemodialysis duration, Evolution, Endocrinology, Medical research, Nephrology

## Abstract

This study was aimed to investigate the evolution of bone mineral density (BMD) in patients with maintenance hemodialysis (MHD) by using phantom-less quantitative computed tomography (PL-QCT). We collected patients with MHD in Suzhou Hospital of Nanjing Medical University from September 2020 to December 2023 as the prospective observation group. BMD of thoracolumbar vertebra, total hip and femoral neck were measured by PL-QCT. Patients with MHD were divided into 9 groups according to hemodialysis duration. Chest CT scans of patients in prospective observation group were collected during the first three months of MHD and 1 year, 2 years, 3 years after dialysis between January 2017 and December 2023 as the retrospective observation group, and BMD of the twelfth thoracic vertebra was measured. According to the BMD changes among the prospective observation group and the retrospective observation group, the evolution of thoracolumbar vertebral BMD, whole hip BMD and femoral neck BMD were comprehensively analyzed. BMD of thoracolumbar vertebra gradually decreased within 36 months in patients with MHD. Thoracolumbar vertebral BMD tended to increase when hemodialysis duration was more than 36–48 months, and thoracolumbar vertebral BMD increased significantly with hemodialysis duration when hemodialysis duration was more than 60 months, and significantly exceeded the BMD before MHD. BMD of total hip and femoral neck gradually decreased within 36 months in patients with MHD. BMD of total hip and femoral neck increased with hemodialysis duration when hemodialysis duration was more than 72 months, but was almost the same as that of the first year of MHD. In the follow-up evaluation of BMD in MHD patients, it is recommended to use QCT to measure BMD in thoracolumbar vertebrae or hip the first 3 years of MHD, and use QCT to measure BMD in thoracolumbar vertebrae to evaluate changes over 5 years of MHD.

## Introduction

China has the largest number of patients with chronic kidney disease in the world^1^and maintenance hemodialysis (MHD) is the main treatment for patients with end-stage kidney disease. Chronic kidney disease-mineral and bone disorders (ckd-mbd) is a common complication in patients with MHD. It can lead to mineral metabolism disorder, metastatic vascular calcification, increased risk of fractures, sarcopenia, malignant tumor and so on^2^. Therefore, active and rational management of patients with MHD is essential to reduce complications and to improve the quality of life of patients with MHD.

Bone mineral density (BMD) assessment is an important tool for the integrated management of bone health in patients with MHD^3^enabling early detection of bone disorders, fracture risk assessment, monitoring treatment outcomes, and reduction of adverse outcomes associated with bone and cardiovascular disease. BMD assessment has been widely used to diagnose osteoporosis (OP) and predict the risk of fractures in elderly people and postmenopausal women, but it is insufficient to assess BMD in patients with MHD.

Dual-energy X-ray absorptiometry (DXA) and quantitative computed tomography (QCT) are the most important methods for assessing BMD, and previous studies^3–5^ have confirmed that QCT is a similar but more sensitive method for assessing bone microstructure, bone mass change, and predicting fracture risk compared to DXA. QCT has higher three-dimensional spatial resolution, can separate cortical and trabecular bone while DXA cannot, and the bone alterations in the cortical and trabecular bone in patients with CKD are not synchronous^6,7^. Furthermore, current studies^8–10^ showed that QCT could more accurately assess bone status in CKD patients compared to DXA since DXA may overestimate spinal BMD, likely due to the high prevalence in these patients of arthritis, scoliosis, and abdominal aorta calcifications.

Conventionally, the subject must be scanned at the same time as the standard phantom in phantom-based quantitative computed tomography (PB-QCT). However, the standard phantom is susceptible to subtle artifacts caused by the gap between the subject and the standard phantom, and the need to establish correction equations between different imaging devices makes the operation steps of PB-QCT complicated^11,12^. Phantom-less quantitative computed tomography (PL-QCT) uses internal calibration material of patients themselves instead of requiring the phantom to be simultaneously scanned with patients, and the reference value of internal calibration material is determined by multiple CT scanners from different CT manufacturers^13,14^. In addition, PL-QCT can be used for retrospective analysis of chest and abdominal CT.

So far, there are few articles using QCT to study the evolution of BMD in patients with MHD. The prospective studies conducted by Malluche et al.^6,15^ and Lechleitner et al.^16^ investigated the changes in BMD of dialysis patients one or two years after baseline measurement, while they did not conduct detailed grouping based on hemodialysis duration to investigate the changes in BMD at the baseline measurement even the hemodialysis duration of their research subjects exceeded ten years. Besides, Iseri et al.^17^ investigated the changes of BMD measured by DXA during the initial year of hemodialysis and their research results showed that total BMD and BMD of specific parts (such as head, leg, pelvis, spine, etc.) decreased significantly. Brunerová et al.^18^ observed decreased BMD in the lumbar spine, proximal femur and femoral neck two years after hemodialysis by using Lunar Prodigy densitometer. A cross-sectional study of Amirkhanlou et al.^19^ pointed out that when hemodialysis duration exceeded three years, OP was more likely to occur in the whole hip and femoral neck compared with the vertebral body. From this it can be seen, the focus of these articles lies in the evolution of BMD at the early stage of dialysis or with a short time span, and there is a lack of detailed grouping of hemodialysis duration to study the long-term evolution of BMD in patients with MHD.

Therefore, this study is aimed to use PL-QCT for evaluating the evolution of BMD in patients with MHD, with a particular focus on the long-term evolution and more detailed grouping of hemodialysis duration, providing a reference for delaying and managing CKD-MBD and reducing related complications.

## Materials and methods

This study was approved by the Ethics Committee of the Suzhou Hospital of Nanjing Medical University (K-2022-179-K01), and informed consent was obtained from each participant. The research was performed in accordance with the declaration of Helsinki.

### Study design and participants

A total of 156 patients with MHD in the Affiliated Suzhou Hospital of Nanjing Medical University from September 2020 to December 2023 were collected. We excluded 2 cases of double hip fractures, 5 cases of hemodialysis duration less than 6 months, 2 cases of peritoneal dialysis, 5 cases of age lower than 40 years or higher than 85 years, 6 cases of too little fat and 4 cases of muscle CT value lower than 40 HU, and finally collected a total of 132 patients for the prospective observation group. The enrolled patients received routine hemodialysis 3 times a week for 4 h each time, using standard bicarbonate dialysis solution, and the calcium ion concentration of the dialysis solution was 1.5 mmol/L. All patients received standardized management of calcium and phosphorus metabolism. Through continuous monitoring of serum calcium, phosphorus and parathyroid hormone levels, individualized drug adjustments were made according to clinical guidance to ensure the stability of mineral metabolism in patients with MHD in this study.

Chest CTs within the first three months of MHD (pre-dialysis), 1 year of dialysis, 2 years of dialysis, and 3 years of dialysis were also collected from the prospective observation group from January 2017 to December 2023, which was used for follow-up analysis. The BMD of T12 vertebrae were measured. A total of 44 patients with 1 year of follow-up chest CTs, 25 patients with 2 years of follow-up chest CTs, and 13 patients with 3 years of follow-up chest CTs as the retrospective observation group.

### Data acquisition

Clinical data of patients were collected, including age, sex, hemodialysis duration, primary disease, the history of secondary hyperparathyroidism (SHPT), fracture history after hemodialysis, the history of tobacco smoking. All patients underwent Chest and abdominal CT scans, and the examinations were performed on the 64-slice CT scanner (Philips Ingenuity CT, Netherlands) and 128-slice CT scanner (Philips Brilliance iCT, Netherlands). Measurements: During the examination, the subject was placed in a supine position with arms raised above his head and held his breath at maximal inspiration, and kept the toes of both feet together. Scanning parameters: 120 kVp, 100–300 mAs, slice thickness 5 mm and reconstruction thickness 1 mm for chest CT; 120 kVp, 200–500 mAs, slice thickness 5 mm and reconstruction thickness 1 mm for abdominal CT.

### Data processing

BMD was measured by using Bone’s FRAX software, version 1.1.1 (Guangdong, China). Bone’s FRAX software measures BMD by the phantom-less calibration method. It utilizes coarse segmentation, tissue-specific thresholding, and two-dimensional convolution operation to efficiently perform filtering and select the optimal ROI location computed by the proposed automatic method. This series of algorithms makes the automatic selection of fat and muscle ROI more accurate. The normal distribution of CT values can reduce the measurement error caused by manual operation. Compared with PB-QCT, this PL-QCT system has higher precision in the assessment of BMD^20^.

The BMD of the thoracolumbar vertebrae was measured in the T12-L2 vertebrae in the prospective observation group, and the BMD of the remaining vertebrae was measured when either of the T12, L1, or L2 vertebrae was fractured. In the retrospective observation group, only the chest CT of the patients was collected, while the L1 and/or L2 vertebral bodies were not scanned in some patients. We compared the BMD of the T12, L1, L2 vertebral bodies in the prospective observation group. The BMD of the T12, L1, and L2 vertebral bodies in 122 Patients with MHD was finally measured, and the data between the three groups were compared using one-way analysis of variance (ANOVA). The mean T12 spine BMD was (144.89 ± 67.12) mg/cm^3^, the mean L1 spine BMD was (137.38 ± 65.36) mg/cm^3^, the mean L2 spine BMD was (130.13 ± 58.33) mg/cm^3^. However, there was no significant difference in BMD among T12, L1, L2 vertebrae (F = 1.632, *P* = 0.197). Therefore, in the retrospective observation group, the BMD of the thoracolumbar vertebral body was measured in the T12 vertebral body.

**Fig. 1:**
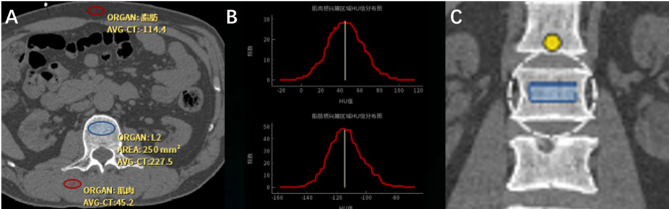
Shows the measurement of thoracolumbar vertebral BMD by using PL-QCT. The blue circle in (**A**) refers to the ROI of the measured vertebral body, The ROI is selected from the cancellous region in the middle of the vertebra to maximize ROI while avoiding the cortical bone, basal vein of the vertebral body, osteophyte and other areas. The red circle indicates the ROI of subcutaneous fat and paravertebral muscle on the same layer of the measured vertebral body. (**B**) shows that vertebral BMD is calibrated by CT values of the ROI of paravertebral muscle and subcutaneous fat on the same level of the measured vertebral body, and the ROI region needs to be adjusted to a normal distribution on the calibration curve. (**C**) shows coronal ROI.

PL-QCT was used to highlight ROI with the same points of attention as PB-QCT, which has been described in previously published articles^20,21^. But BMD measured by PL-QCT was calibrated by CT values of the subcutaneous fat and paravertebral muscle ROI at the same level as the measured vertebral body (Fig. 1). The vertebral BMD measured was averaged to obtain the thoracolumbar vertebral BMD, and the measurements were expressed as mg/cm³.

**Fig. 2:**
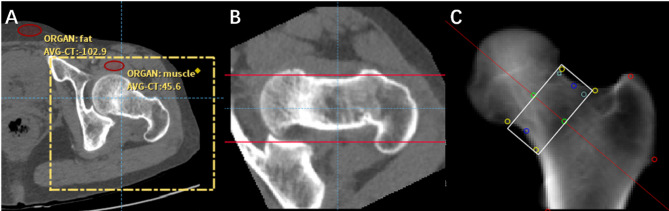
Shows the measurement of whole hip BMD and femoral neck BMD by using PL-QCT. (**A**) shows the ROI of hip muscle and fat in quality control, (**B**) shows the horizontal state of the femoral neck in axial position, and (**C**) shows the generated coronal volume projection image of the hip, with the ROI of the femoral neck in the box.

The hip BMD was measured on either side of the hip. The ROI of hip muscle and fat should be selected and the quality control curve should be adjusted to normal distribution. The hip should be cut so that the positions of femoral neck and femoral shaft in axial, sagittal and coronal positions meet the requirements: the axial section is when the femoral neck is in a horizontal position, the coronal position is similar to DXA scan images or hip X-ray plain films, and the sagittal position is when the femoral shaft is in a vertical state (Fig. 2). Measurements were expressed as g/cm^2^.

### Statistical analysis

SPSS 27.0 was used to analyze the data. Measurement data were expressed as (‾x ± s). Single-factor analysis of variance (ANOVA) and Kruskal-Wallis H test were used to compare groups. Counting data were expressed as frequency and percentage, and Chi-square test was used for comparison between groups. The collected follow-up data were tested by repeated measures analysis of variance (ANOVA). *P* < 0.05 was considered statistically significant.

## Results

### Clinical data of patients in the prospective observation group

A total of 132 patients were finally enrolled in this study, with an average age of (61.48 ± 10.48) years, an average hemodialysis duration of (93.16 ± 73.88) months. There were 71 males (53.8%) and 61 females (46.2%), and 41 patients (31.1%) with diabetes mellitus. There were 112 patients with hypertension (84.9%), 25 patients with smoking (18.9%), 87 patients with SHPT history (65.9%), and 15 patients with low energy fracture (11.4%). The primary disease was chronic glomerulonephritis in 43 cases (32.6%), diabetic nephropathy in 23 cases (17.4%), hypertensive renal damage in 18 cases (13.6%), polycystic kidney disease in 15 cases (11.4%), and other unknown causes in 33 cases (25.0%).

### The changes of BMD with Hemodialysis duration in the prospective observation group

**Fig. 3:**
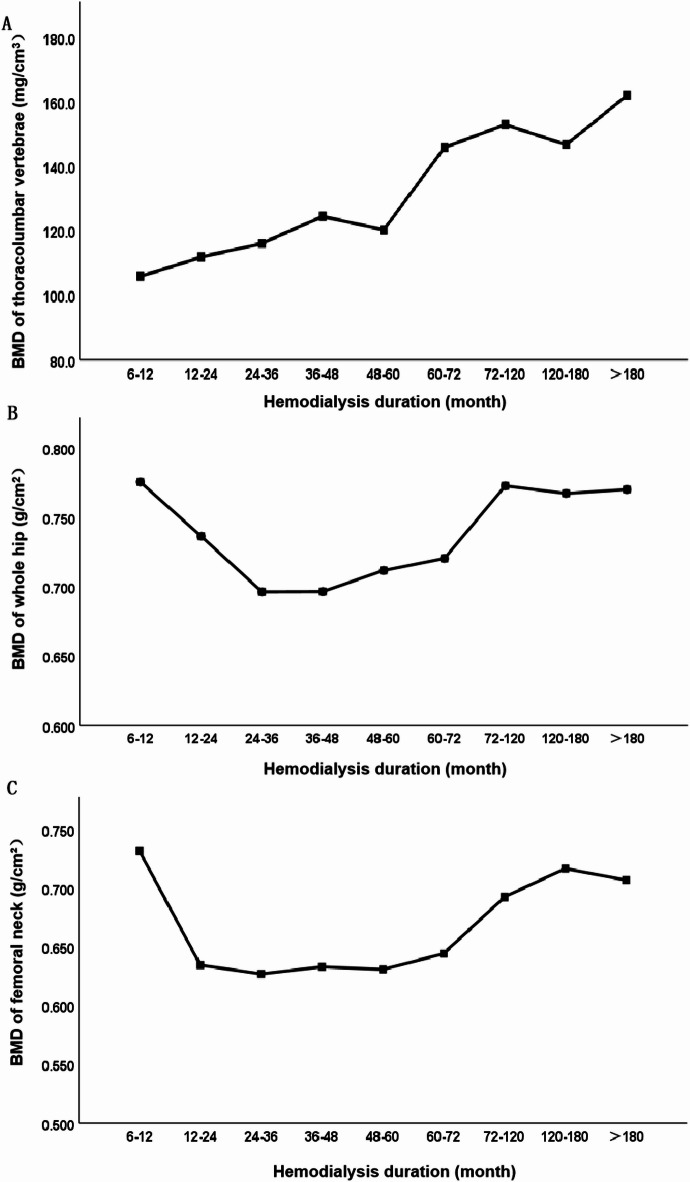
Shows the changes of thoracolumbar vertebral BMD, whole hip BMD and femoral neck BMD in patients with MHD over hemodialysis duration. As shown in (**A**), BMD of thoracolumbar vertebrae showed a gradual upward trend after 36–48 months of dialysis. After 60 months of hemodialysis duration, BMD of thoracolumbar vertebrae increased significantly with hemodialysis duration and was significantly higher than the initial stage of dialysis. (**B**,**C**) showed that BMD of the whole hip and femoral neck gradually decreased within 36 months of dialysis, and increased with hemodialysis duration after 72 months of hemodialysis duration, but was almost the same as that in the first year of dialysis.

**Fig. 4:**
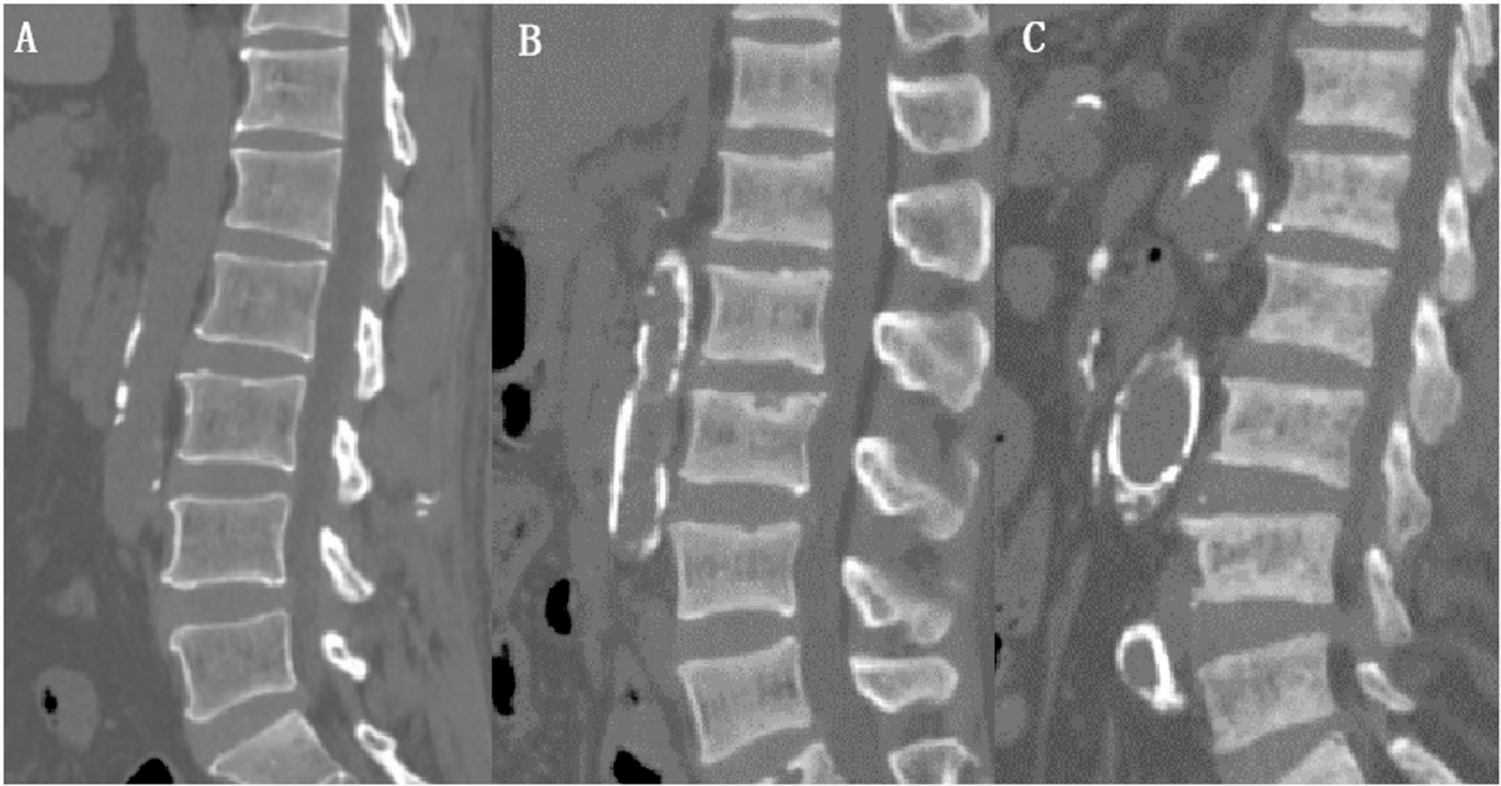
Shows the changes of thoracolumbar vertebral BMD in patients with MHD over hemodialysis duration. (**A**) shows a male, 58 years old, with a BMD of 117.0 mg/cm^2^ after 18 months of dialysis; (**B**) shows a male, 60 years old, with a 77-month dialysis, with a BMD of 154.6 mg/cm^2^; (**C**) shows a female, 66 years old, with a 179-month dialysis, with a BMD of 233.1 mg/cm^2^. The BMD of thoracolumbar vertebrae increased significantly after 60 months of hemodialysis duration, and the BMD was significantly higher than that at the initial stage of dialysis.

**Fig. 5:**
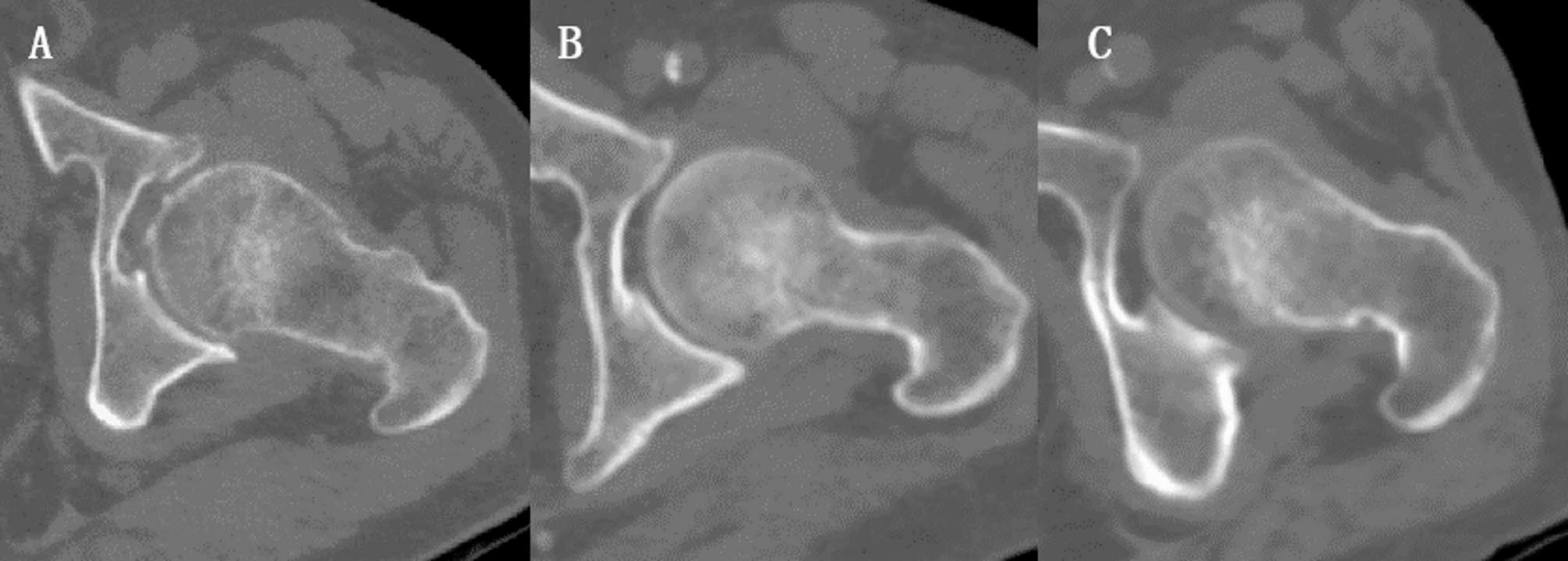
Shows the changes of whole hip BMD and femoral neck BMD in patients with MHD over hemodialysis duration. (**A**) shows a male, aged 66, with a BMD of whole hip and femoral neck of 0.736 and 0.634 g/cm^2^ after 28 months of dialysis; (**B**) shows a female, aged 65, with BMD of whole hip and femoral neck of 0.766 and 0.670 g/cm^2^ after 72 months of dialysis; (**C**) shows a female, aged 60, with a dialysis of 183 months. The BMD of the whole hip and femoral neck were 0.797 and 0.722 g/cm^2^, which increased with hemodialysis duration but were almost the same as that at the initial stage of dialysis.

The 132 patients with MHD were divided into 9 groups according to the hemodialysis duration: 6–12, 12–24, 24–36, 36–48, 48–60, 60–72, 72–120, 120–180, and > 180 months. There were no differences in age and gender between the groups (*p* = 0.589 and 0.224). Although there were no statistically significant differences in the BMD of thoracolumbar vertebrae, whole hip and femoral neck among the 9 groups, there was a trend of gradual increase in the BMD of thoracolumbar vertebrae after 36–48 months of dialysis, and the BMD of thoracolumbar vertebrae increased significantly after 60 months of hemodialysis duration and significantly exceeded that of the initial stage of dialysis. The BMD of whole hip and femoral neck gradually decreased within 36 months after dialysis, and increased with hemodialysis duration after 72 months of hemodialysis duration, but the BMD was almost the same as that in the first year of dialysis. The BMD distribution of thoracolumbar vertebrae, whole hip and femoral neck in patients with MHD with over hemodialysis duration was shown in (Table 1, Figs. 3, 4 and 5).

**Table 1:** The changes of BMD with Hemodialysis duration in the prospective observation group.

Hemodialysis duration(month)/*n*	Thoracolumbar vertebral BMD (mg/cm^3^)	BMD of whole hip (g/cm^2^)	BMD of femoral neck (g/cm^2^)
6–12(*n* = 10)	105.78 ± 27.54	0.78 ± 0.12	0.73 ± 0.12
12–24(*n* = 19)	111.79 ± 42.47	0.73 ± 0.19	0.63 ± 0.18
24–36(*n* = 9)	116.07 ± 37.21	0.70 ± 0.09	0.63 ± 0.09
36–48(*n* = 12)	124.48 ± 49.35	0.70 ± 0.20	0.63 ± 0.17
48–60(*n* = 12)	120.24 ± 42.84	0.71 ± 0.23	0.63 ± 0.19
60–72(*n* = 8)	145.82 ± 64.38	0.72 ± 0.17	0.64 ± 0.16
72–120(*n* = 13)	152.94 ± 64.91	0.77 ± 0.22	0.69 ± 0.15
120–180(*n* = 24)	146.68 ± 60.53	0.77 ± 0.20	0.72 ± 0.17
>180(*n* = 25)	161.97 ± 90.26	0.77 ± 0.28	0.71 ± 0.23
P	0.090	0.951	0.584

### Analysis of changes in BMD of T12 vertebrae in the retrospective observation group

In the prospective observation group of 132 cases, we collected the data of the patients before dialysis, 1-year after dialysis, 2-year after dialysis and 3-year after dialysis as retrospective observation group. A total of 44 patients with chest CT scans within 3 months before MHD (before dialysis) and 1-year after dialysis were collected, with an average age of (65.57 ± 10.96) years, including 26 males (59.1%) and 18 females (40.9%). Chest CT scans of 25 patients with before dialysis, 1-year and 2-year after dialysis were collected, with an average age of (63.12 ± 12.34) years, including 13 males (52.0%) and 12 females (48.0%). Chest CT scans of 13 patients with before dialysis, 1-year, 2-year and 3-year after dialysis were collected, with an average age of (60.38 ± 11.38) years, including 6 males (46.2%) and 7 females (53.8%).

The BMD of T12 vertebrae decreased gradually within 3 years of dialysis. It is particularly worth noting, the BMD at 1-year after dialysis was lower than that before dialysis (*p* < 0.001), the BMD at 2-year after dialysis was lower than that before dialysis (*p* = 0.009). However, there was no statistical difference between the BMD at 2-year after dialysis and that at 1-year after dialysis (*p* = 0.712), and there was no statistical difference between the BMD at 3-year after dialysis and that at 2-year after dialysis (*p* = 0.726). The BMD changes of T12 vertebrae in the retrospective observation group were shown in (Tables 2 and 3).

**Table 2:** The changes of BMD of T12 vertebrae in the retrospective observation group.

Follow-up time (year)/*n*	BMD of T12 vertebrae (mg/cm^3^)			
	Before dialysis	1-year after dialysis	2-year after dialysis	3-year after dialysis
1-year(*n* = 44)	127.32 ± 36.28	115.18 ± 32.42	–	–
2-year(*n* = 25)	131.00 ± 35.49	121.98 ± 31.29	119.95 ± 41.23	–
3-year(*n* = 13)	135.75 ± 38.40	127.17 ± 35.99	124.64 ± 38.41	123.08 ± 35.13

**Table 3:** The comparison of BMD of T12 vertebrae in the retrospective observation group.

	Before – 1-year	Before – 1-year	Before – 2-year	1-year - 2-year	Before – 1-year	1-year - 2-year	2-year - 3-year
	*n* = 44	*n* = 25			*n* = 13		
F	17.062	6.852	8.198	0.140	4.742	0.135	0.129
P	<0.001	0.015	0.009	0.712	0.050	0.720	0.726

## Discussion

Previous studies have discussed the changes of BMD in the initial stage of hemodialysis. Iseri et al.^17^ showed that in the first year of hemodialysis, total BMD and BMD of specific parts (such as head, leg, pelvis, spine, etc.) measured by DXA decreased significantly, with a decrease of 1.54% in pelvic BMD and 1.86% in spinal BMD. Brunerová et al.^18^ observed significant annual loss of BMD and trabecular bone score in patients within the first two years of MHD, with BMD of lumbar decreasing by 4.1%, BMD of total hip decreasing by 9.1%, and BMD of femoral neck decreasing by 1.3%. Our study showed that the changes of BMD in the thoracolumbar vertebrae, total hip, and femoral neck at the initial stage of MHD were similar to those reported in the previous literature.

Our results also showed that BMD in the thoracolumbar vertebrae, total hip, and femoral neck was elevated in patients with long-term MHD, especially those who had been on dialysis for more than 5–6 years. Previous studies have shown that with the extension of hemodialysis duration, patients with MHD are more likely to show decreased BMD due to mineral metabolism disorders, SHPT and other factors affecting bone turnover, mineralization and volume changes^19,22,23^. The results of our study were inconsistent with most previous reports, but the results of Zhan et al.^24^ showed that the average BMD of patients with MHD was higher than that of the normal control group, especially in patients with MHD who had SHPT, which was consistent with our conclusion. The study of Sladowska et al.^25^ indicated high BMD of vertebral bones in MHD patients from their dialysis department, and their BMD was similar to that observed in healthy volunteers. We speculate that the BMD of patients with long-term MHD increases over hemodialysis duration. First, the occurrence of renal bone disease in patients with long-term MHD leads to decreased bone circulation and bone resorption, which may paradoxically result in increased BMD but weakened and brittle bones^26^. Second, dialysis-related amyloidosis may increase BMD. Onishi et al.^27^ found that beta 2-microglobulin accumulation was rarely seen in patients who had been on hemodialysis for less than 6 years, but 19% of patients who had been on hemodialysis for more than 10 years had beta 2-microglobulin accumulation, which may lead to amyloid deposition in bone, potentially increasing BMD. Third, complications such as SHPT in dialysis leads to increased use of vitamin D analogues, phosphate binders and other drugs, and BMD can be improved and increased with the increase of the application of therapeutic drugs and treatment time^28,29^. Fourth, studies have shown that BMD of spine and hip in patients with diabetes^30,31^ is increased compared with that of normal people, which may be due to the retention of trabecular bone microstructure parameters caused by hyperinsulinemia. In addition, high body mass index in patients with diabetes can increase the mechanical load of bone, and the increase of visceral adipose tissue can increase pro-inflammatory cytokines and stimulate osteoclast factors, which is manifested as increased cortical porosity and increased BMD.

Although patients with long-term MHD show an increasing trend of BMD, this does not always translate into a healthier skeletal state, because part of the trabecular bone formation is at the expense of the cortex of bone, which is very harmful to bone strength, and we should pay more attention to changes in bone quality and the risk of fractures in these patients^32,33^.

In addition, our study found that the increase in BMD was earlier and more pronounced in the thoracolumbar vertebrae than in the hip in patients with long-term MHD. The bone is composed of cortical and trabecular bone, with a high proportion of cortical bone in the proximal femur or femoral neck, and cortical thickness is a changeable structural property of the hip joint. Therefore, aging and complications such as SHPT lead to accelerated cortical bone absorption, continuous thinning of the cortex, and increased cortical porosity in patients with MHD, which are ultimately prone to hip fracture. Malluche et al.^6^ used QCT to evaluate hip bone changes in patients with MHD during two-year follow-up. During hip BMD measurement, the BMD of the whole hip, cortical bone and trabecular bone were measured according to the BMD of single voxel compared with the threshold of 350 mg/cm³ and the partial volume compensation algorithm to adjust cortical volumes. The results showed that the BMD, cortical bone mass and volume of the whole hip measured by QCT decreased significantly (-5.9%, -7.3% and − 10.0%) within 2 years, and the trabecular bone volume increased (5.9%). The increase in trabecular bone volume may be due to the transformation of cortical bone into trabecular bone. In contrast, trabecular bone, the three-dimensional reticular structure of vertebrae, changes more rapidly than cortical bone because it makes up 80% of the bone surface^2^. In a previous study using bone histology, 630 patients with end-stage renal disease were found to have increased cortical porosity, trabecular thickness and BMD due to overactivity of parathyroid hormone^34^. The ROI measured by QCT is only for the trabecular bone in the middle of the vertebral body, so it is easier to detect increased BMD on the thoracolumbar vertebrae in patients with long-term MHD.

The study had several limitations. First of all, the sample size of this study was small, which may limit the general applicability of our findings, and future multi-center and large sample studies may be needed. Second, the number of cases in the retrospective observation group was small, and the observation period was short, so further prospective and longer observation period was needed. Third, based on the results of this study, further studies will discuss the value of factors such as the regulation of clinically relevant blood indicators and treatment methods in delaying and managing CKD-MBD.

## Conclusion

In patients with MHD, the BMD of thoracolumbar vertebra, total hip and femoral neck showed a characteristic trend of decreasing first and then increasing, with the BMD of thoracolumbar vertebrae increasing significantly after 60 months of dialysis and significantly exceeding the pre-dialysis BMD, and the BMD of total hip and femoral neck increasing after 72 months of dialysis, but was almost the same as the BMD in the first year of dialysis. In the follow-up evaluation of BMD in patients with MHD, it is recommended to use QCT to measure BMD in thoracolumbar vertebrae or hip the first 3 years of MHD, and use QCT to measure BMD in thoracolumbar vertebrae to evaluate changes over 5 years of MHD.

## Data Availability

The data underlying this article will be shared on reasonable request to the corresponding author.
